# Occurrence of genetic modifications in core, 5′UTR and NS5b of HCV associated with viral response to treatment

**DOI:** 10.1186/1743-422X-11-171

**Published:** 2014-09-30

**Authors:** Sobia Kanwal, Tariq Mahmood

**Affiliations:** Department of Animal Sciences Faculty of Biological Sciences, Quaid-i-Azam University, Islamabad, 45320 Pakistan; Department of Zoology, PMAS University of Arid Agriculture Rawalpindi, Rawalpindi, Pakistan; Department of Plant Sciences, Faculty of Biological Sciences, Quaid-i-Azam University, Islamabad, 45320 Pakistan

**Keywords:** HCV genetic heterogeneity, HCV response to treatment

## Abstract

**Background:**

It is becoming progressively more understandable that genetic variability of viruses is a major challenge in translating the laboratory findings to clinic. Genetic variability is the underlying cause of variant viral proteins which are not targetable by host immunological machinery.

**Methods:**

500 patients were enrolled in study and amongst them, 451 patients were followed and categorized into two groups on the basis of their treatment response. Group 1 consisting of the 376 patients exhibited SVR while group 2 comprised 75 patients who were non-responders on the basis of viral load as evidenced by Real-Time PCR. Comparative sequence analysis was done between 75 non-responders and 75 responders (randomly picked from 376) by targeting three genomic regions, 5′UTR, core and NS5B and amplified products were directly sequenced and obtained sequences were cleaned, aligned and submitted to GenBank. Maximum Parsimony (MP) method was used for phylogenetic analysis and dendrograms were dragged using MEGA 5. Heterogeneity at nucleotide and amino acid level was determined using software BioEdit and DNAman while phosphorylation and N-linked glycosylation sites were determined using NetPhos 2.0 and SignalP-NN.

**Results:**

Genotype 3 was prevalent in group 1 whereas non-responders indicated rare genotypes of Pakistan i.e. 4 and 5, genotype 6q and 6v were reported first time from Pakistan in this study. At nucleotide and amino acid level, the genetic distance and mutation, number of predicted N-phosphorylation and N-glycosylation sites was higher in group 2 as compared to group 1. Difference in percentage composition of individual amino acids was noted to be different between the two groups.

**Conclusions:**

It can be concluded that heterogeneity both at nucleotide and amino acid level contributed in developing drug resistant phenotype. Moreover, occurrence of rare genotypes might hurdle the way to positive response of conventional treatment. Furthermore, prediction of phosphorylation and glycosylation sites could help in targeting the proper sites for drug designing.

## Background

HCV has emerged as one of the most extensively and deeply studied molecular pathogen reported to be involved in different diseases including hepatocellular carcinoma (HCC), liver cirrhosis and end-stage liver disease [1]. High throughput technologies have started to shed light on recently broadening signaling landscapes reported to be modulated by HCV. Substantial fraction of scientific information has been added into the existing pool of molecular virology and it is now known that HCV reconstitutes signaling machinery of host cells to control multifaceted biological mechanisms.

It is the only known member of the genus hepacivirus belong to the family flaviviridae. It can transmit through blood transfusion, organ transplantation, drug injection, dental exposure, body tattoos, and sexual exposure or through vertical transmission. Size of HCV genome is approximately 9.6 kb. The highly conserved RNA structures at 5′ untranslated region (5′ UTR) contained the internal ribosome entry site (IRES) and at 3′ untranslated region (3′ UTR) contained stable stem loop and an internal poly (U)-poly (U/C) tract [2]. UTRs flanked single Open Reading Frame (ORF) encoding a polyprotein of about 3000 amino acids [3, 4] which after processing produced 11 mature proteins. In the endoplasmic reticulum (ER) of host cell some cellular and viral proteases cleaved a single polypeptide protein thus producing mature structural and nonstructural regulatory proteins [5]. Structural proteins encapsulate the viral nucleocapsid and comprise the core (C) and two envelope (E1 and E2) proteins. Six nonstructural (NS) proteins included NS2, NS3, NS4A NS4B, NS5A and NS5B [6]. Structural proteins can be separated from nonstructural protein on the basis of short membrane peptide p7 (which is assumed to be a viroporin). Rapid evolutionary rate of HCV results in alarming genetic diversity and leads to generation of six different genotypes (1–6) which further branched into subtypes, e.g. 1a, 1b, 1c etc. [7].

Antibodies production after the viral infection counteracted viral replication. Genetic variability has been noted to play role in chaperoning virus from immunosurveillance [8]. Besides other factors, the genotype difference play a crucial role in viral response to treatment, as the interaction of certain HCV proteins with intracellular biochemical pathway such as NS5A protein coding region, E2 and intracellular pathways might mediate the effects of IFN [9]. The association of sequence diversity of the viral NS5A to IFN responsiveness has also been described [10].

Production of a quasispecies (closely related but genetically distinct variants) permits HCV to escape from host defense and antiviral therapies. At least 90% nucleotide sequence homology resulted in two quasispecies [11]. Therefore, HCV sensitivity to therapy is uneven as this 10% of genetic divergence can possibly create various viral variants with a diverse response towards treatment. Moreover, HCV patients with small quasispecies sequence are more prone to attain SVR as compared to patients exhibiting high complexity and significant changes in the composition of quasispecies. Alteration in some subgenomic regions of HCV has been associated with IFN treatment sensitivity. Moreover, any mutation in amino acid sequence of IFN sensitivity determining region (ISDR) [12] and IFN/ ribavirin resistance–determining region (IRRDR) is considerably related with higher SVR rates. These two regions are important to play a role in treatment response that’s why any mutation in these two regions might influence the response to IFN therapy.

Viral adaptation leads to viral diversification which further results in viral escape from immune system. HVR-1 in E2 region of virus genome has been reported as dominant neutralization epitope and its carboxy-terminal possesses epitopes for T-helper cells and other cytotoxic response [13]. The presence of immune pressures may also help to distinguish maternal viral clones passed to child and this may also control the completion of the viruses in both the mother and child over time. Other factors involved might be the mode of transmission and potentially different quasispecies populations [14].

Here in the present study three genomic regions of HCV are being focused in response to the therapy. The study will also unravel the role of genetic heterogeneity and the genetic modifications occuring in the concerned genomic regions of virus to escape the immune response.

## Results

### Seroepidemiology of HCV positive patients in response to the treatment

**Figure 1:**
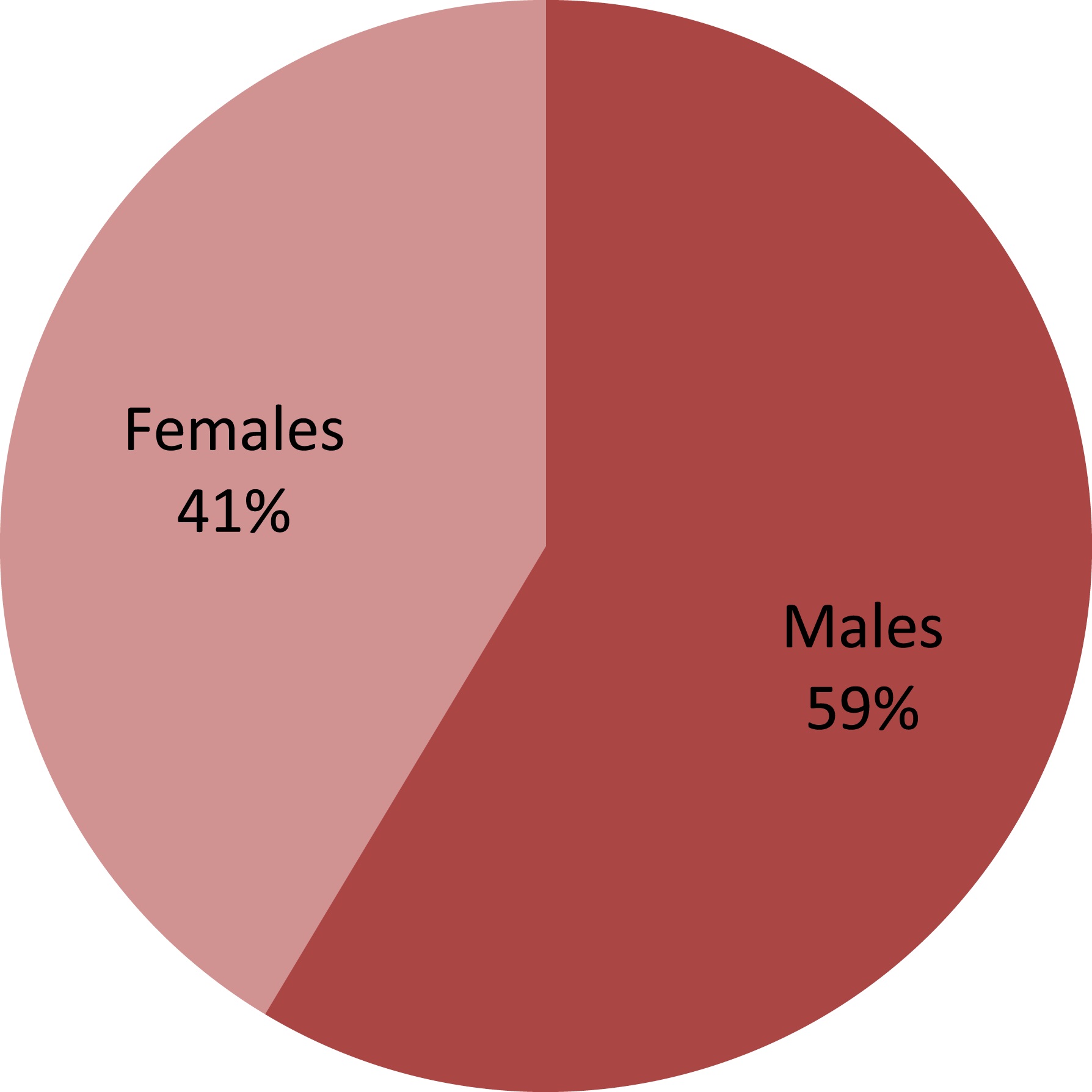
**Gender distribution of HCV positive patients in the present study.**

The data was collected on HCV from Pakistan institute of medical sciences Islamabad (PIMS), combined military hospital (CMH) Rawalpindi, military hospital (MH) Rawalpindi and Fauji Foundation hospital (FFH) Rawalpindi from the patients visiting the liver OPDs with different ethnic origins from all over the Pakistan. Initially 500 patients gave consent to participate in the study. Among them 207 were females while 293 were males. Overall the ratio of the HCV positive males was high as compared to females (Figure 1) in the present study.

### Natural history of the HCV for the studied cohort

**Figure 2:**
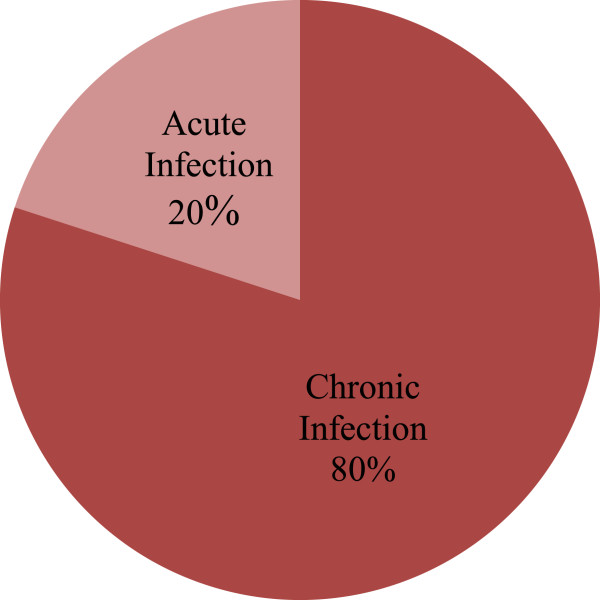
**Distribution of patients on the basis of chronicity of disease.**

The selected cohort of patients constituted of 80% chronic cases of HCV and only 20% acute infection. There was no apparent gender differences in the rate of chronicity in hepatitis C infection. Majority of the considered cases whether males or females were equally suffering from the chronicity of the disease (Figure 2).

### Determination of viral load

**Figure 3:**
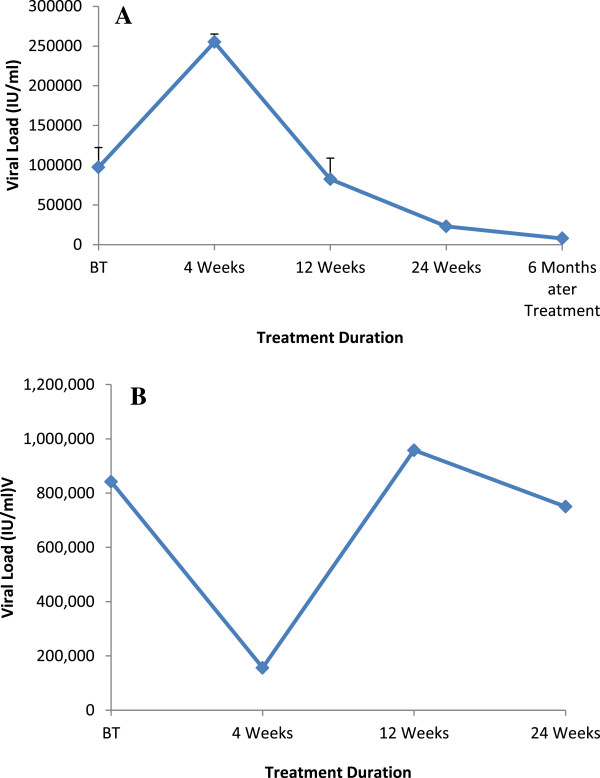
**Graphical representation of HCV viral load during different treatment periods in both the treatment groups. (A)**: Group 1 (with SVR) **(B)**: Group 2 (non-responders).

Quantities of HCV RNA, in IU/ml, were determined by comparing the results for the unknown serum samples to those on a standard curve. In group 1 (SVR) the baseline viral load was low as compared to the group 2 (non-responders). Univariate analysis revealed that patients in group 1 (with SVR) possessing low baseline viral load showed significant decline in viral RNA copies at the start of therapy (P > 0.000) while become completely HCV RNA negative and remain so afterwards even when analyzed 6 months after the end of therapy. However, patients with high baseline viral load took 24 weeks treatment to become completely HCV RNA negative. Furthermore in group 2 (non-responders), patients did not show any significant decline in their viral load till 24 weeks of treatment (P = 0.076) (Figure 3).

### Genotype distribution among patients

**Figure 4:**
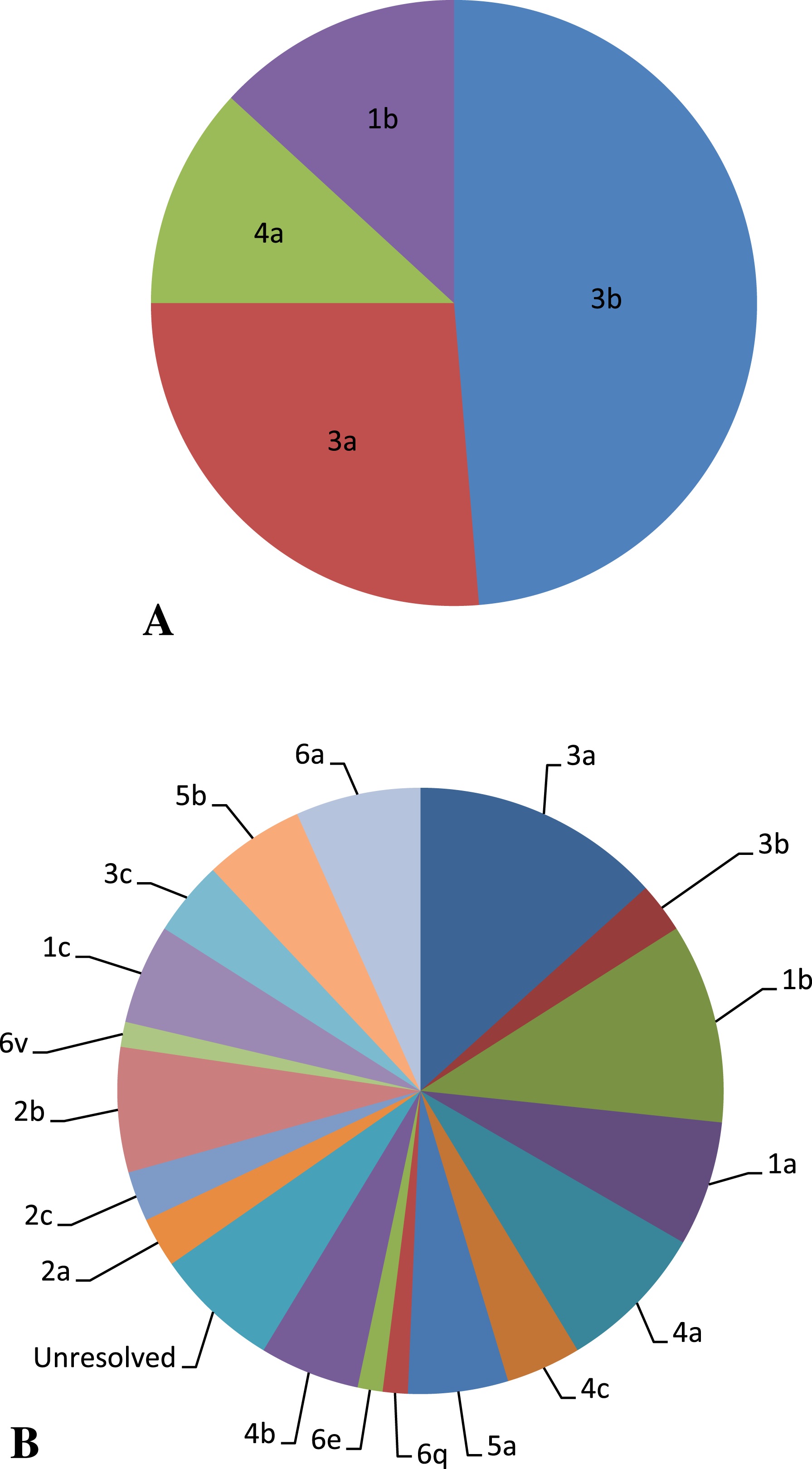
**Frequency distribution of HCV genotypes. A**: in group 1; **B**: in group 2.

Similar genotypes were resolved on the basis of sequence analysis of all the three genomic regions core, 5′UTR and NS5B. In group 1 genotype 3 was present in 74.6% of patients while 13.3% and 12% of patients showed the genotype 1 and 4 respectively. However in group 2 that constituted the non-responders genotype 1 was most prevalent one and revealed by 22.6% of the patients while the second most prevalent genotype is genotype 3 exhibited by 20% of the patients. Genotype 4 was displayed by 17.3% of patients while equal distribution of genotypes 5 and 6 of about 10.66% were explored. Genotype 6q and 6v have been reported for the first time from Pakistan in this study. Moreover genotype 2 was present in 12% of patients in addition only 6.66% patients remain untypeable (Figure 4).

### Phylogenetic analysis

**Figure 5:**
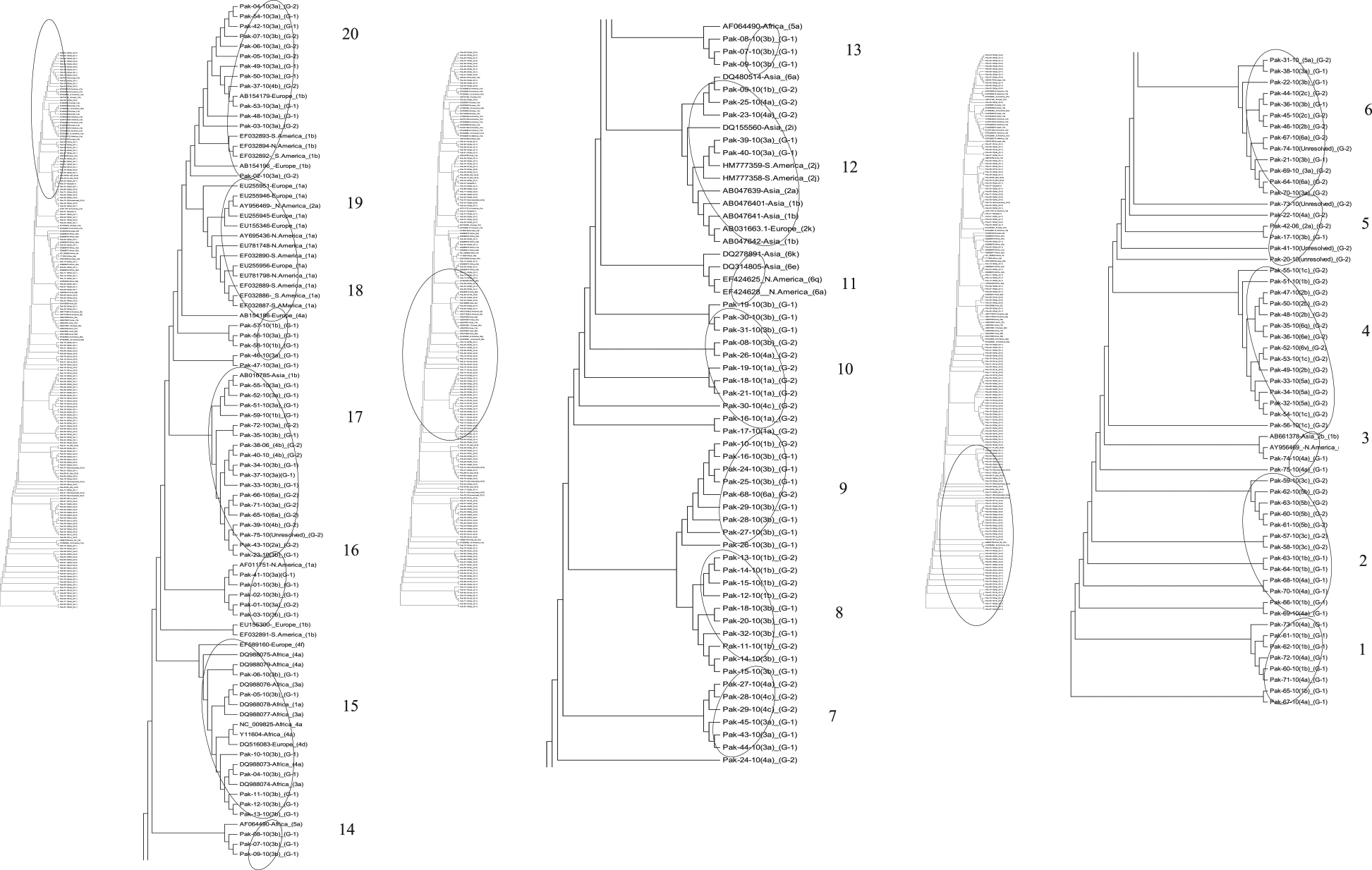
**Parsimonious phylogenetic tree constructed using 5′UTR sequences of patients from both groups with reference sequences from database.**

**Figure 6:**
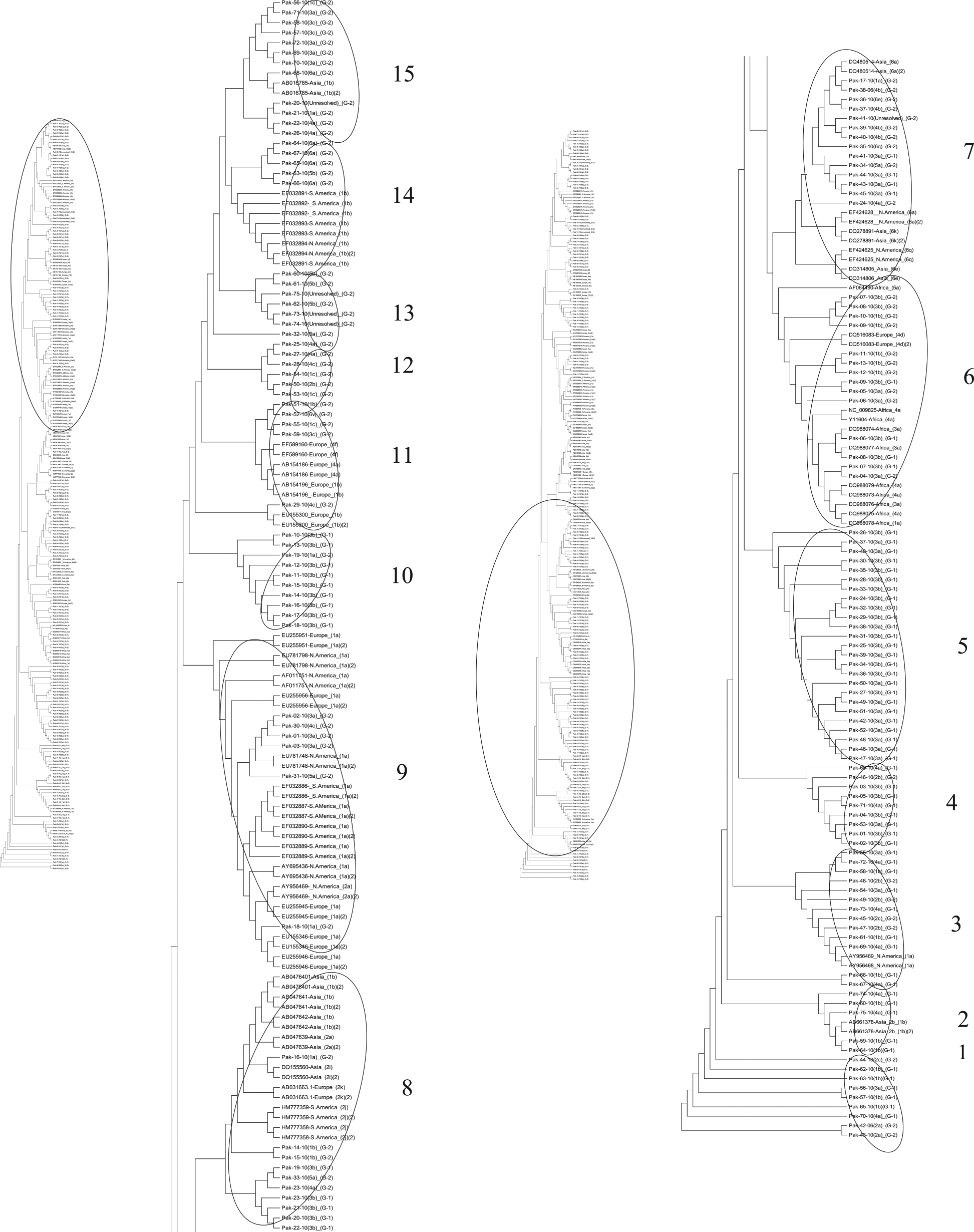
**Parsimonious phylogenetic tree constructed using core sequences of patients from group both groups with reference sequences from database.**

**Figure 7:**
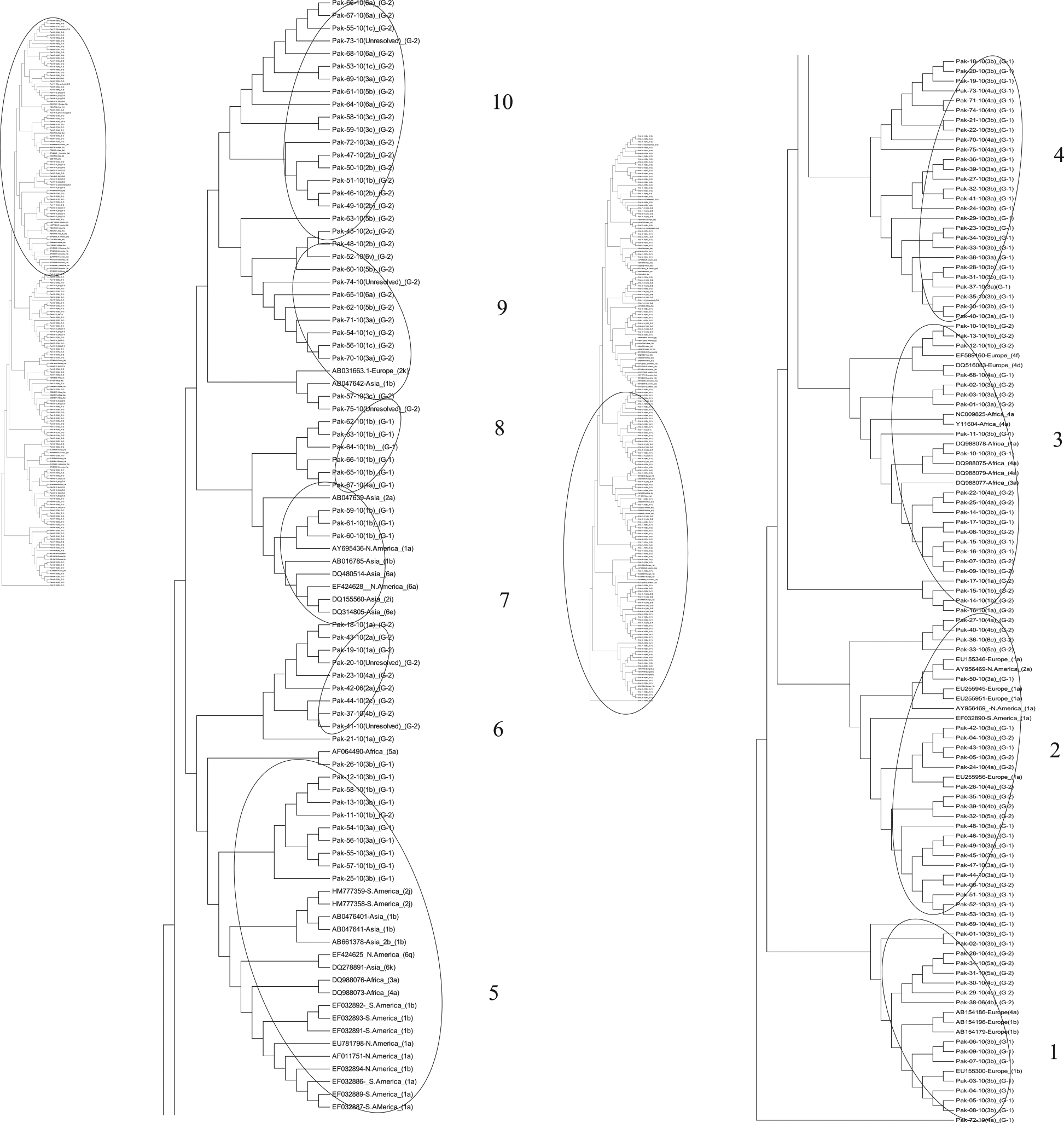
**Parsimonious phylogenetic tree constructed using NS5B sequences of patients from group both groups with reference sequences from database**.

Phylogenetic tree constructed maximum parsimony method (MP) using sequences of 5׳ UTR from both treatment groups exhibiting the mix pattern of evolution of both the treatment groups (Figure 5). Overall the tree was divided into 16 clusters. Cluster 1 and 2 constitute some sequences of group 1 dipicting the early evolution of the sequences from patients who responded positively to therapy. Moreover sequences from group 1 were present in all the clusters of the tree. Majority of the sequences from the group 2 were present in the recently evolved clusters (clusters 12, 13, 14, 15, 16, 19 and 20). Most of the sequences from group 2 make their separate clusters while somewhere in the tree they possess the position along with the reference sequence from the database (clusters 6, 7, 8, 9, 10, 12). The unresolved genotypes were found in close neighbor to genotype 2, 3, 4 and 6 (clusters5, 6 and 17).Evolutionary tree based on core sequences exhibited the similar branching pattern of group 1 and 2. Overall the tree was divided into 15 clusters. Some sequences from group 1 and 2 were found at the base of the tree and exhibited the early evolutionary period (cluster 1). In cluster 2 sequences from group 1 showed close homology with Asian isolates. At some positions throughout the tree topology some of the sequences from both groups were clustered with each other (clusters 4, 5, 10, 12 and 13) while in others clusters they were found in close proximity with reference strains throughout the world (clusters 3, 6, 7, 8, 9, 11, 14 and 15). Three out of 5 unresolved genotypes are present in cluster 13 and in neighbor of genotype 5 while one unresolved strain was found in cluster 15 along with the genotype 1and another unresolved strain took the place in cluster 7 along with genotype 4 (Figure 6).Dendrogram constructed using sequences from NS5B exhibited diverge branching pattern by almost all the sequences from both groups 1 and 2. Tree was divided into 10 clusters. Sequences from group 1 and 2 along with the reference sequences from Europe were positioned at the base of the tree exhibiting the early evolutionary behavior. Most of the sequences from group 2 were depicting the recent evolution (cluster 6, 8, 9 and 10). Some sequences from group 1 and 2 presenting the homology with sequences from Africa, America (North and South) and Europe (cluster 2, 3, 5, 7 and 8). Moreover the unresolved sequences showed homology with genotypes 1, 3, 4 and 5 and 6 (cluster 6, 8, 9 and 10) (Figure 7).

### Nucleotide sequence diversity of 5′ UTR, CORE and NS5B

It was observed that patients who responded treatment (SVR) had significantly (P = 0.003, P = 0.005) less number of transitions and genetic distances than the non-responders (Table 1). It is also shown that the total number of mutation spots (including all the insertions, deletions, transitions and transversions) were less among responders (group 1) than in the non-responders (group 2) (P = 0.007).

**Table 1:** **Genetic diversity of all the three genomic regions (5′ UTR, core and NS5B) of HCV isolates for both treatment groups**

Factors	5′ UTR			Core			NS5B		
	Group 1 (n = 75)	Group 2 (n = 75)	P Values	Group 1 (n = 75)	Group 2 (n = 75)	P Values	Group 1 (n = 75)	Group 2 (n = 75)	P Values
**Insertions**	0.33 ± 0.52	1.08 ± 1.9	< 0.001	0.23 ± 0.521	0.05 ± 1.1	< 0.001	0.34 ± 0.52	1.18 ± 1.9	< 0.001
**Deletions**	0.66 ± 0.82	1.2 ± 0.59	< 0.002	0.65 ± 0.22	1.1 ± 0.53	< 0.001	0.61 ± 0.20	1.0 ± 0.10	< 0.030
**Transitions**	1.8 ± 0.9	4.7 ± 2.0	< 0.001	1.7 ± 0.4	4.1 ± 1.0	< 0.003	2.0 ± 0.7	4.8 ± 1.6	< 0.015
**Transversions**	2.0 ± 2.5	2.5 ± 2.1	< 0.051	1.9 ± 0.5	2.4 ± 0.1	< 0.001	1.8 ± 0.3	1.8 ± 1.1	< 0.073
**No. of mutation spots**	6.2 ± 3.1	8.2 ± 4.2	< 0.043	3.1 ± 0.3	7.2 ± 4.0	< 0.002	9.2 ± 2.1	11.2 ± 4.2	< 0.004
**Genetic distance**	0.005 ± 0.24	0.13 ± 0.75	< 0.001	0.007 ± 0.31	0.11 ± 0.21	< 0.003	0.001 ± 0.25	0.17 ± 0.27	< 0.001
**Non-synonymous substitution**	0.07 ± 0.21	0.09 ± 0.13	< 0.050	0.007 ± 0.10	0.07 ± 0.03	< 0.001	0.15 ± 0.11	0.35 ± 0.14	< 0.031
**Synonymous substitutions**	0.005 ± 0.20	0.006 ± 0.01	< 0.053	0.001 ± 0.02	0.01 ± 0.01	< 0.031	0.004 ± 0.12	0.006 ± 0.12	< 0.030

Group 1: Patients with SVR.

Group 2: Non-responders.

The targeted genomic regions (5′ UTR, core and NS5B) in the present study also showed more non-synonymous substitutions than synonymous in group 2 (non-responders) as compared to group 1 (with SVR). Present results showed the occurrence of more variability in sequences of NS5B as compared to the sequences of 5′ UTR and core. Our findings showed that nucleotides 24–170 of 5′ UTR are highly conserved between the two studied groups as compared to the other two genomic regions (Table 2).

**Table 2:** **Conserved and variable regions of HCV in the nucleotide sequence of three genomic regions (5′ UTR, core, NS5B) among the two groups**

Treatment groups	5′UTR		P values	Core		P values	NS5B		P values
	Conserved region	Variable Region		Conserved region	Variable region		Conserved region	Variable region	
Group 1 (n = 75)	30-70, 70–84, 90–105, 124–140, 153-170	4-29	<0.001	70-91, 100–114, 120–142, 224-254	20-39, 143–169 320-341	<0.043	134-144, 353–370, 400-419	20-40, 60–87, 90–111, 420–439, 510–520, 600-619	<0.003
Group 2 (n = 75)	32-54, 61–80, 81–101, 120–135, 136–145, 150–164, 200-223	1-31, 104-119	<0.001	23-45, 67–91, 111–124, 222–252,	1-20, 254–267, 300–320, 354-378	<0.64	24- 38, 134- 151	1-20, 100–131, 200–237, 500-532, 610–624, 650-672	<0.001

### Frequency of amino acids

**Figure 8:**
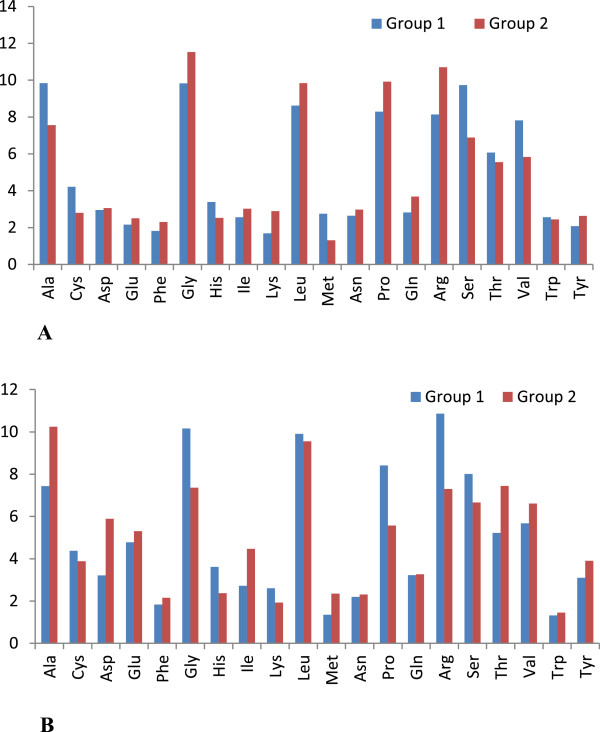
**Comparison of amino acid frequency between the two groups; (A) Core and (B) NS5B.**

The amino acid sequences of core and NS5B were aligned and frequency of amino acid was determined using MEGA 5. Percentage of the individual amino acids in both core and NS5B were compared between the two groups (1 and 2). Some of the amino acids were equally frequent among all the samples while some showed the considerable divergence among the two groups. Amino acids, alanine, glycine, proline, arginine and serine showed the remarkable difference between the core sequences of the both groups (Figure 8A). When the amino acid sequences of the NS5B were compared between the both groups that showed a considerable change in amino acids i.e., alanine, aspirgilin, glycine, histidine, isoleucine, proline, arginine and threonine (Figure 8B).

### Analysis of amino acid heterogeneity

**Figure 9:**
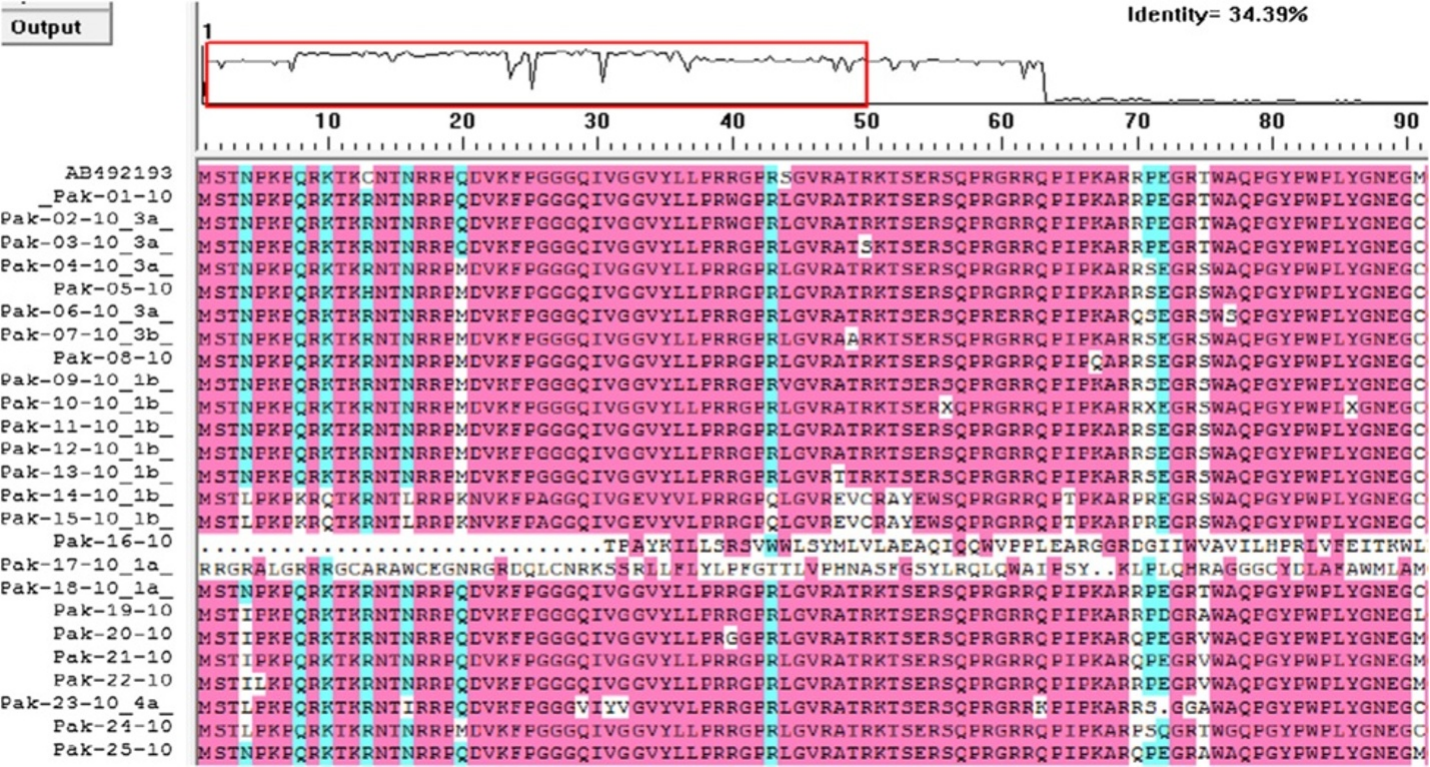
**Representative output of DNAman.** Amino acid substitution in the study sequences in comparison to the reference sequences obtained from DNAman.

The output of the MEGA 5 and DNAman revealed that total number of conserved and variable sites was considerably different in both treatment groups with the difference in amino acid positions. The conserved amino acid positions in SVR patients showed substitution in amino acids in non-responders (Table 3). Multiple amino acid substitution at single site was more common in non-responders than in SVR patients for example at position 1 and 4 amino acids histidine and threonine were replaced by four other amino acids in non-responders whereas in SVR patients at these positions single substitutions were observed. Graphical output of some of the amino acid substitution is given in the Figure 9.

**Table 3:** **Conserved and variable regions of HCV in the amino acid sequences of core, NS5B among the two groups**

Treatment groups	Conserved amino acids positions		P value	Variable amino aqcids positions		P value
	Core	NS5B		Core	NS5B	
Group 1 (n = 75)	3, 9, 10, 15, 21, 23, 25, 28, 35, 105, 106, 123, 134, 136, 137, 142	14,18,65,120,133,141	< 0.001	24, 29, 34, 36, 38, 39, 41, 65, 72, 85, 112,141, 151	35, 43, 44,47, 48, 51, 59, 62, 65, 68, 70, 87, 89, 95, 102, 107, 112, 118, 120, 129, 221, 240, 256	0.002
Group 2(n = 75)	3, 7, 9, 25, 28, 35, 106, 165, 205	13, 18, 24, 67, 85, 109, 120, 134, 143, 154, 153	< 0.001	23, 29, 35, 37, 38, 41, 43, 54, 56, 67, 72, 78, 85, 105, 112, 120, 135, 141, 156	26, 35, 48, 95, 105, 109, 108, 114, 118, 132, 143, 154, 165, 163, 172, 187, 201, 221, 240, 289, 267,256	< 0.03

### Prediction of N-linked phosphorylation site

**Figure 10:**
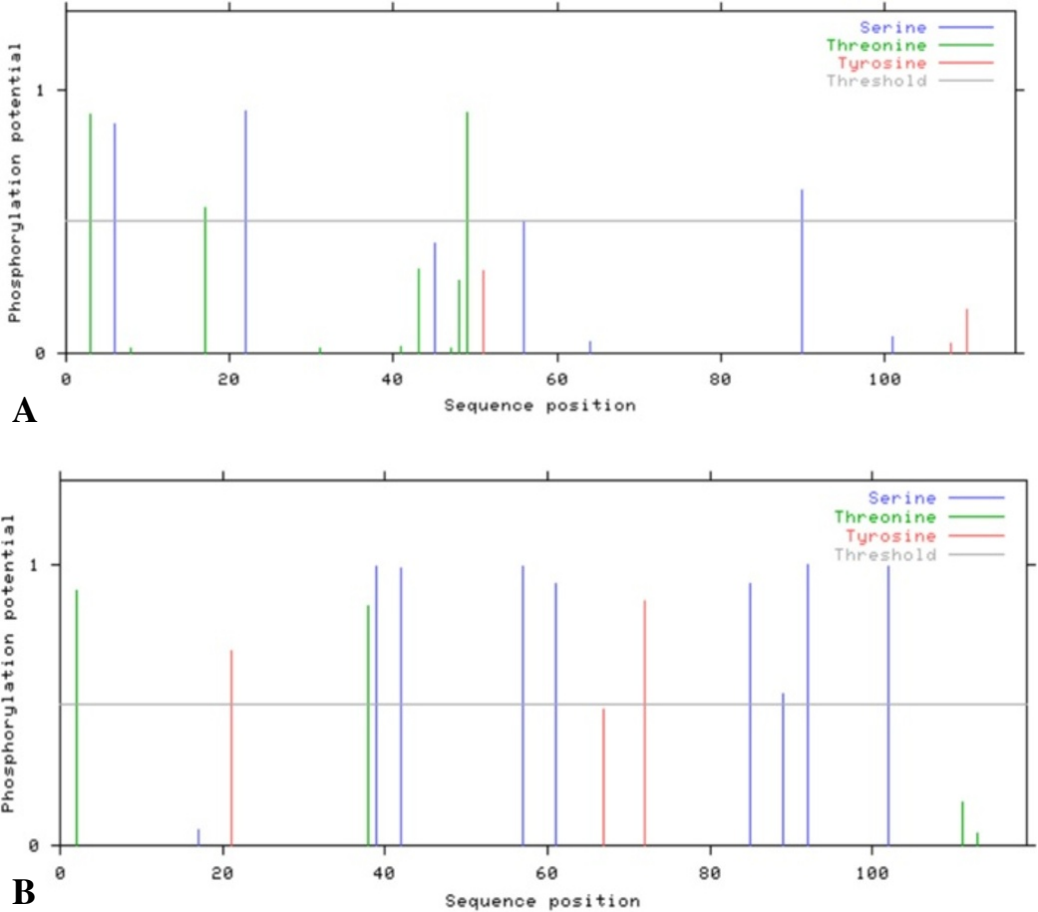
**Phosphorylation sites predicted in the core protein sequences for both treatment groups (A) group 1 (with SVR); (B) group 2 (non-responders).**

**Figure 11:**
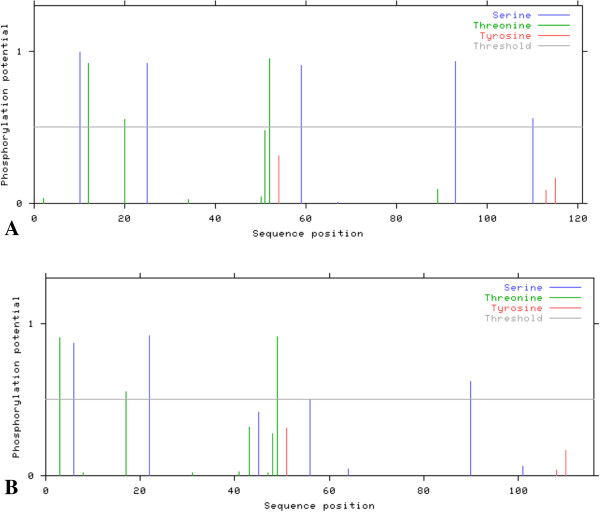
**Phosphorylation sites predicted in the NS5B protein sequences for both treatment groups (A): group 1 (with SVR); (B): group 2 (non-responders).**

*In silico* N-linked phosphorylation sites were predicted using NetPhos server 2.0. It was observed that amino acid sequences of core protein in non-responders (group 2) exhibit more potential phosphorylation sites as compared to those shown by sequences of patients with SVR (group 1).Moreover the amino acid sequences of NS5B did not show any considerable difference in number of predicted phosphorylation sites between the two groups. Representative predictions among the 75 sequences each for both groups and also for both proteins are given in Figure 10 and 11. Predictions were made on the “Low Stringency” to identify as many sites as possible.

### Determination of N-linked glycosylation sites

**Figure 12:**
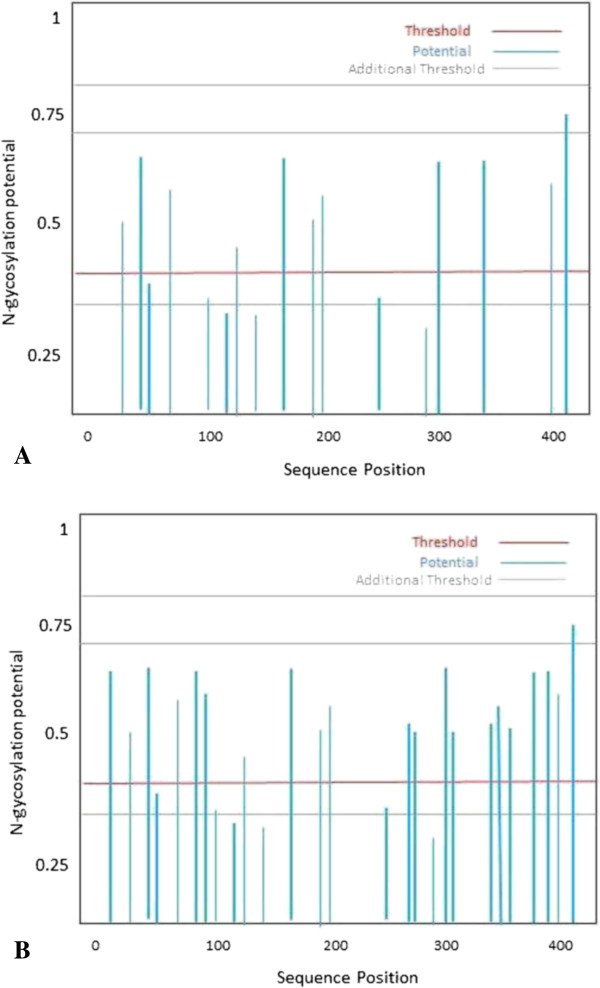
**Potential N-glycosylation sites in core protein sequence predicted from SignalP-NN: (A) group I (with SVR); (B) group 2 (non-responders).**

**Figure 13:**
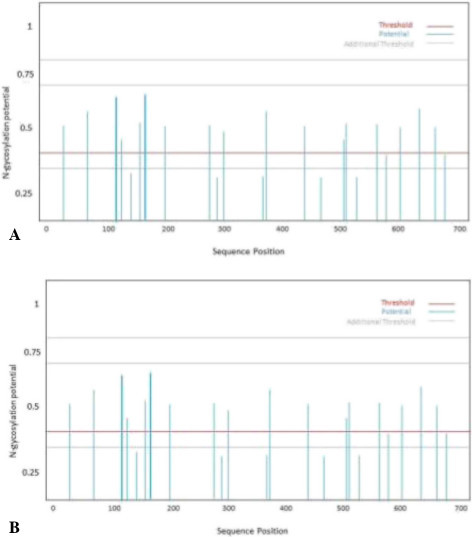
**Potential N-glycosylation sites in NS5B sequence predicted from SignalPNN: (A) group I (with SVR); (B) group 2 (non-responders).**

Increase in N-linked glycosylation sites was observed in core and NS5b protein sequences in patients who did not respond to the therapy (group 2) as compared to those who showed SVR (group 1) (Figure 12 and 13).

## Discussion

### Seroepidemiology of HCV positive patients in response to the treatment

In the present study high ratio of the HCV positive males was observed as compared to females. Majority of the previous studies reported from all over the world including Pakistan, where the number of male patients was greater than females [15–18]. It is already known that progression of HCV infection is worse in males than in females [19, 20]. Independent of alcohol intake, females have twofold lesser progression rate to fibrosis compared with males [21]. In males higher rate of HCV prevalence is possibly due to exposure to various risk factors. This trend might be due to the fact that males particularly from Pakistani social setup are more exposed to the HCV risk factors i.e. blood transfusions, dental procedures, barber shears, needle sick injuries and tattooing as compared to female patients. According to our cultural environment there is trivial exposure of females to some of the risk factors e.g. tattooing, injection drug use, barbers etc. Moreover our cultural trait associates the higher prevalence in males.

### Natural history of the HCV for the studied cohort

In the current study the selected cohort of patients constituted of 80% chronic cases of HCV and only 20% acute infection. These results are in accordance with the ones previously reported by Al-Moslih and Al-Huraibi [22] from Yamen, where the majority of the reported cases were chronic and the number of acute cases was 19.4%. Major reason supposed to be involved in this chronicity pattern is that the HCV infection is asymptomatic in 75–80% of the cases. It has been studied that only 20–25% of the patients report to the hospitals with some presenting complaints [23]. However, in Pakistan a common man does not prefer to visit physician for routine checkup, which might leads to chronicity of disease.

There were no apparent gender differences in the rate of chronicity in hepatitis C infection. Majority of the considered cases whether males or females were equally suffering from the chronicity of the disease. Similar observations were reported from other studies around the world, such as chronicity was related to age and the estimated rate was 30% and 76% in subjects’ ≤ 20 and ≥ 20 years of age, respectively [24, 25].

### Determination of viral load

In the present study viral load was quantified as the predictive factor for HCV response to treatment. Patients in group 1 possess low baseline viral load that dropped significantly with the start of therapy. This is steady with the previous report where poor response to standard antiviral therapy was observed in patients with higher viral load in contrast to patients having lower viral load [26]. The degree of HCV infection has been associated with increased viral load [27].

Many investigators reported that baseline viral load has been linked with low rates of response to standard interferon therapy [28–30]. Therefore, it is shown that along with HCV genotypes, baseline viral load also act as predictor of response to antiviral therapy [31]. Several studies have shown positive response to currently available antiviral therapy in patients with lower baseline viral load (<80,0000) in comparison to high pre-treatment viral load (>80,0000) [32–34]. Similar findings also suggest that a decline in HCV viral load during the first 2–12 weeks of antiviral therapy is an indicative of prognostic therapeutic efficacy [35, 36]. Therefore, HCV baseline viral load, decrease of viral load during initial phase of therapy and genotype exhibited important roles in altering and improving antiviral treatment [37]. Herein non-response to the therapy might be due to high baseline viral load, further there must be some additional host and viral factors that would be responsible for the viral response to the treatment.

### Genotype determination

Genotypes of HCV are important predictors for duration of antiviral therapy, choice of interferon and probability of SVR [38–40]. In the present study, number of patients exhibiting genotype 3 (a and b) is more in group 1 (SVR) as compared to group 2 (non-responders) describing the positive response of genotype 3 to antiviral therapy. Previously, it was also established that genotype 3 is associated with short treatment duration and also with less cost and side effects [41]. Several authors described HCV genotype 3 (a and b) as the most prevalent genotype in Pakistan [42], [43–46]. Moreover, different reports revealed that the number of patients with HCV genotype 3 are increasing with time in Pakistani population [47–50]. Hence an increased rate of HCV genotype 3 in Pakistan is a good anticipation for treatment as well as control of HCV infection [51, 52]. Furthermore, the prevalence of this genotype in Pakistani population established its predominance in the neighboring countries i.e. India [51, 53], Iran [54], Bangladesh [51, 55] and China [53]. It is quite possible that genotype 1 may have entered into Pakistan from these countries through local persons crossing borders for job and trade.

Genotypes 2, 5 and 6 were not displayed by patients in group 1 whereas non responders showed their presence. Genotype 2 is also prevalent in Pakistan [56]. This genotype is considered as interferon responsive [57] while in the present study patients possess the HCV genotype 2 belongs to group 2 (non- responders). Genotype 2 is prevalent in Europe where 3% and 9% in Spain and France respectively of HCV patients were diagnosed as infected with genotype 2. Other than Europe, a high frequency of HCV genotype 2 has been reported in some Southern African and Asian areas where it accounts for 30% of chronic HCV infection overall [58].

Genotype 6q and 6v have been reported for the first time from Pakistan in this study. The few published studies on treatment involving HCV genotype 6 generally suggest that genotype 6 behaves more similar to genotypes 2 and 3 [59, 60] and responds better to therapy than genotype 1. The number of patients possessing genotype 4 is high in non-responders in the current study; this might be due to the fact that previously this genotype has been considered difficult to treat because initial clinical trials using conventional IFN-α monotherapy produced limited effect [61]. Furthermore, genotype 4 is reported to be associated with liver cirrhosis [62]. In actual genotype 4 is the most important and prevalent strain of Egypt [63], North Africa and the Middle East [64, 65]. Some studies from Pakistan supported the presence of genotype 4 in Pakistani population and reported prevalence is 3% [47] and 2.48% [49] in the blood samples of Pakistani population. One possible reason for the existence of this genotype in Pakistan might be the neighboring country Iran where Genotype 4 is a prevalent genotype which may be due to its geographical location near to Europe and Middle East [66, 67]. Previous studies showed that HCV genotype 5 appears to be an easily treatable virus, with response rates compatible with those of genotypes 2 and 3 after a 48-week course of therapy [68, 69]. This is contradictory to our study where it showed the resistance to antiviral therapy. Genotype 5 is prevalent only in South Africa [69] and is very rare in Asia while a reports from Pakistan which declared the presence of genotype 5 in this region [55]. It is investigated that rate of viral response to therapy in patients with HCV genotype 5 was high, regardless of the patients’ advanced age, high pretreatment virus loads, and high level of liver fibrosis [41]. As these factors are known to obstruct the viral response to treatment.

This genotype was initially reported in Africa [65]. In group 1 (SVR) genotypes of all the samples were resolved while among patients of group 2 (non- responders) 5 sample remained unresolved. Unresolved genotypes might be caused by the mutations [69, 70] and these mutations may be either point mutations (Transitions and transversions) or they may be insertions, inversions, or deletions and translocations etc. The inability of HCV to perform proof reading and its high mutation rate both have made it genetically successful according to Darwinian theory of natural selection [71]. The present study explored the presence of some uncommon genotypes that could be the possible reason of non-response to the antiviral therapy.

### Determination of phylogeny

To track the role of evolutionary behavior of the 5′UTR, core and NS5B in viral response to therapy, the dendrograms were constructed using studied sequences with respect to the reference sequences downloaded from database. The mix evolutionary pattern was shown by all the three genomic regions of the study and recent evolution was shown by sequences of patients from group 2. A general trend in the dendrograms indicates that actively mutated and newly evolved sequences were mainly unresolved. The reason of them being unresolved is the high mutation rate of HCV [72, 73] as different geographical and epidemiological patterns determine the genotypes and subtypes of HCV [74, 75]. Studied strains showed the similarity with Asian, European and American (North and South) strains, might be due to some migration events. A study describes that there is a sizeable community of South Asians like Asian labor migrants settled in European countries. Some of the present study strains showed homology with African stain and they lie in the same cluster with African strains. Currently, approximately 2 million South Asians are living in Africa; some came in late nineteen and early twentieth century [76]. Similarly, a study showed that South American countries are populated with European, Asian and Africans [77]. A limited migration pattern has been identified among strains of Europe, North America, South America and Africa that have shown high diversity in their respective geographical regions as reported previously that in areas of endemicity a highly divergent pattern was observed among the strains suggesting long infection duration [78]. Some strains did not show any branching pattern giving an idea about absence of any change that has occurred in them for years. This might be because of the high negative selection pressure which they are undergoing due to some environmental factors whereas their non-response to the therapy might be due to some other change at nucleotide or amino acid level or some host factors involved. Therefore, here it can be proposed that less diversification leads to positive response towards antiviral therapy.

### Nucleotide sequence diversity of 5′ UTR, CORE and NS5B

Overall the nucleotide sequence of 5′UTR, core and NS5B showed more diversity in group 2 as compared to group. Increase in homogenous viral population associated with viral clearance is a result of intense reduction in genetic diversity independent of genotype. Mutation rate determines the viral ability to keep basic information while for any given genome; mutation rate determines the ability of a virus to maintain essential information while surviving with different environmental changes [79, 80]. The higher rates of non-synonymous mutations in the group 2 as compared to group 1 are also considered to modify the epitopes and help the virus to evade both the treatment and immune system. Present results showed the occurrence of more variability in sequences of NS5B as compared to the sequences of 5′ UTR and core. It is evident that genomic regions such as the 5′ UTR and the core are highly conserved; the non-structural regions NS2, 3, 5b and the 3′ UTR are relatively variable, while the envelope regions E1 and E2, NS4 and the NS5A genes displayed the maximum sequence diversity [81]. Increased variability of NS5B also showed encoded the RNA-dependent RNA polymerase which is error prone and deficient in proofreading and resulted in random introduction of base changes into the viral genome [82]. This become more problematic for the infected patient, as quasispecies variation confers significant adaptive potential on HCV and has been concerned in the evasion and control of the host response to infection and in differential sensitivity to IFN therapy. The hostile antiviral host environment may drive the proliferation of HCV “evasion variants” from a pre-existing quasispecies pool or through viral genetic adaptation [83].

### Frequency of amino acids

Remarkable difference was observed in amino acids; alanine, glycine, proline, arginine and serine between the core sequences of the both groups. Many studies have reported that substitutions in the HCV core region results in enhanced insulin resistance, steatosis, oxidative stress and HCC [84, 85]. The phylogenetic analysis depicts that viral genome undergone various significant changes with time at different rates in which core region is considered to be more diverse [46]. The study of sequence diversity in HCV core region has additional value since this viral protein has been related with several elements potentially involved in HCV pathogenesis and persistence [86].

Amino acids i.e., alanine, aspirgilin, glycine, histidine, isoleucine, proline, arginine and threonine showed a considerable change in sequences of NS5B between the two groups. This observation is supported by a number of *in vitro* studies that have identified key positions within NS5B that can dramatically affect HCV replication [87]. Moreover, a correlation has been demonstrated between the number of amino acid substitutions in NS5B and replicative capacity [88, 89] as well as HCV treatment outcome [90].

Thus, increased mutations within NS5B may have a deleterious effect on viral replication both *in vitro* and *in vivo*. The existence of multiple unique NS5B variants within an infected individual permits rapid viral adaptation to immunologic and antiviral selection pressures and the cellular microenvironment.

### Analysis of amino acid heterogeneity

Amino acid mutations cause the changes in protein phenotype and thus considered as most deleterious. Though, some of these substitutions are neutral when the protein function does not affected by mutation and are maintained by genetic drift. Both the genomic regions concerned in the present study both are important in the stability of HCV genome and its replication. Earlier it has also been investigated that amino acid substitution in the HCV core region could be a useful predictor of the virological response to peg-IFNα plus RBV combination therapy [91]. Moreover as NS5B is error prone and lacking the proofreading, therefore any change resulted in random introduction of base leads to changes in the viral genome [82] that might affect it response towards therapy. Therefore the RdRp is an important target for the development of anti-HCV drugs [92–95]. Hence, estimation of any change in these genomic regions would be helpful in determining the further strategies against HCV.

### Prediction of N-linked phosphorylation site

Presence of more of potential phosphorylation sites in amino acid sequences of core protein was observed in group 2 as compared to group 1 while no difference was observed in the amino acid sequences of NS5B between the two groups. It is well known that the first defence line against viral infection in mammals is IFN system [96]. Viral particles replicate inside the host cell and infect the surrounding cell on getting released. However, infected cells also inform the neighboring cells about the viral presence by releasing interferone. In response, the neighboring cells produce ample amount of PKR, an enzyme which is a serine/threonine kinase present in cells in their dormant state, induced by interferon and activated upon autophosphorylation. It plays an important role in cellular antiviral defence as well as in apoptosis, signal transduction and transformation [97]. Ample evidence is available for the important role of PKR in the antiviral effect of interferon [98, 99].

HCV has modified itself to avoid the antiviral effects of PKR [100, 101] and here in the present study it is supposed that IFN resistance may be due to phosphorylation of core proteins. Because HCV core binds to and interacts with PKR and this interaction might be a general phenomenon, regardless of HCV genotype and strain [102]. The current study was also focused on detecting such potential phosphorylation sites in our local isolates of HCV who do not respond to therapy. It is further proposed that this interaction might be responsible for HCV resistance to antiviral therapy which could be further confirmed by dephosphorylating these sites and analyzing its effects on PKR binding and inhibition.

### Determination of N-linked glycosylation sites

Viruses used glycosylation pathways by attaching with N-linked oligosaccharides. Folding and trafficking of envelop and other surface proteins with the help of host cellular chaperones and folding factors are promoted by N-glycosylation. The complexity of viral glycoproteins is amplified by the addition or deletion of glycosylation sites during the period of viral evolution. Any alteration in glycosylation sites effect dramatically the survival and transmissibility of the virus and can alter folding and conformation and finally affecting portions of the entire molecule [103, 104]. Variations in glycosylation alter the interaction with receptors and cause a virus to be more recognizable by the innate factors of host immune cells and less recognized by antibody, thus impacting viral replication and infectivity.

Many viruses (influenza, HIV, hepatitis and West Nile virus) that impact human health use glycosylation for important functions in pathogenesis and immune evasion [105]. Therefore here we can assume that resistant behavior of HCV isolates might be due to the increased glycosylation that protect the viral particle to respond to the therapy.

## Conclusions

It can be concluded that viral heterogeneity both at nucleotide and amino acid level contributed equally in resistance to therapy. Besides this, appearance of rare genotypes is also one of the important factors that hurdle the way to positive response of conventional treatment. Computational analysis showed that genetic diversity, any change or mutation in core region, NS5B and 5′UTR might be the cause of HCV strains to resist IFN therapy. Furthermore, prediction of phosphorylation and glycosylation sites could help in targeting the proper sites for drug designing.

## Materials and methods

### Ethical statement

All the research work and experimental protocols were approved by Local Ethics Committee of the Department of Animal Sciences Faculty of Biological sciences Quaid i Azam University Islamabad and whose guidelines were strictly followed. This study was also approved by institutional review board Quad i Azam University Islamabad.

### Patients selection

Overall 451 HCV ELISA positive patients were selected for the study of age group between 20–65 years from Pakistan institute of medical sciences Islamabad (PIMS), combined military hospital (CMH) Rawalpindi, military hospital (MH) Rawalpindi and Fauji Foundation hospital (FFH) Rawalpindi from the patients visiting the liver OPDs by taking the informed written consent (consent was also approved by the institutional review board). Patients with history of alcohol, or positive for HBV or HIV antibody were not considered for the study. All patients were receiving injections of PegIFN-α once each week plus oral RBV daily for 24 weeks as prescribed by the physician. Before the start of therapy viral load was determined in all patients and blood was aspirated and stored for genetic analysis. During the treatment period some of the patients were eliminated and the remaining patients were grouped for further analyses. All the 451 patients were followed till the end of therapy. The study was performed in case control manner. Two groups were defined at the end of the therapy on the basis of their treatment response: group 1 including the patients who exhibited SVR and group 2 constitutes the patients who were non-responders. Among the 451 patients 376 exhibited the SVR while 75 patients remain non-responders. Out the 376 patients with SVR blood sample of 75 patients were randomly picked and proceeded for further analysis.

### Collection of demographic data

Demographic data of all the 500 patients was collected that include age, gender, history of disease, educational levels, occupations, daily work activity (sedentary or non-sedentary), and health behaviors (smoking and alcohol consumption) etc.

### RNA extraction cDNA synthesis and amplification

In the current study three genomic regions including 5′UTR, core and NS5B have been analyzed for their role in response to antiviral therapy in HCV infected patients. Primers were designed in nested manner with external and internal sets for each genomic region in HCV genome (Table 4). The extracted RNA (10 μl) was reverse transcribed into complementary DNA (cDNA) with reverse transcriptase enzymes like Maloney Maurine Leukemia Virus (M-MLV reverse transcriptase enzyme) (Fermentas). Nested PCR was performed by using external and internal set of respective primers of all three genomic regions under consideration in this study along with negative and positive control (already known positive HCV sample).

**Table 4:** **Nucleotide sequences of the designed primers**

PCR	Genomic region	Sequence of primers
Nested	5′UTR	SP1: 5′CTGTGAGGAACTACTGTCTT 3′
		ASPI : 5′ATACTCGAGGTGCACGGTCTACGAGACCT 3′
		SP2: 5′TTCACGCAGAAAGCGTCTAG 3′
		ASP2: 5′CACTCTCGAGCACCCTATCAGGCAGT 3′
Nested	Core	SP1: 5′ ACTGCCTGATAGGGTGCTTGC 3′
		ASP1: 5′ ATGTACCCCATGAGGTCGGC 3′
		SP2: 5′ AGGTCTCGTAGACCGTGCAC 3′
		ASP2: 5′ CACGTAAGGGTATCGATGAC 3′
Semi nested	NS5B	SP1: 5′ GCCTTCACGGAGGCTATGAC 3′
		ASP1: 5′ GGCACCCAAGCTTTCTGAG 3′
		ASP2: 5′ ACACGCTGTGATAAATGTC 3′

### Sequencing and sequence homology

The amplified Products of nested PCR were directly sequenced on Beckman Coulter CEQ 8000 sequencer followed by purification using PCR product purification kit from Genomed. Basic Local Alignment Search Tool (BLAST) was used to find regional homology among all the studied sequences and already reported sequences (http://blast.ncbi.nlm.nih.gov/Blast.cgi). The obtained sequences of the present study were pasted in FASTA format and searched for maximum similarity with already reported HCV sequences.

### Sequence submission to genbank

Sequence data is being submitted to GenBank and accession numbers awaited.

### Determination of phylogeny

The obtained sequences for each genomic region of both treatment groups were aligned separately by ClustalW and similarity of sequences with already reported sequences in database (http://blast.ncbi.nlm.nih.gov/Blast.cgi) was found by Nucleotide Blast (nBlast). Base statistical robustness was performed by 500 bootstrap repeats and the whole process was developed by MEGA 5 software [106]. Multiple aligned sequences were uploaded in MEGA5 and maximum parsimony method was used to construct phylogenetic tree of all the three concerned genomic regions of the present study in comparison to the reference sequences obtained from database from the whole world.

### Sequence diversity of 5′UTR, CORE and NS5B

An online available tool BioEdit version 7.3 was used to determine the sequence diversity at nucleotide level. Rate of insertions, deletions, transversions and transitions in the nucleotide sequences were determined using this software. Besides this mutation spots and number of synonymuous and non-synonymous substitutions were also calculated.

### Translation of nucleotide sequences into amino acid sequence

Nucleotide sequences were required to be converted into amino acid sequences for further analysis. Online available tool TRANSLATE was used for translation (http://hcv.lanl.gov/content/sequence/TRANSLATE/translate.html).

### Analysis of amino acid heterogeneity

Amino acid heterogeneity analysis was done by aligning amino acids of both core and NS5B using Bioedit Clustal W programme. These aligned sequences were then analyzed using MEGA version 5 [83]. Number of conserved sites, number of variable sites and amino acid composition was calculated for each sample of both groups and then comparisons were made between the two groups. Amino acid substitution was determined using online available software DNAman.

### Determination of N-linked phosphorylation site

Online available web server NetPhos 2.0 was used that produces neural network predictions for serine, threonine and tyrosine phosphorylation sites in proteins. Phosphorylation sites were predicted for both the proteins core and NS5B for all the 75 patients each from both groups (1 and 2).

### Determination of N-linked glycosylation sites

To predict the N-glycosylation sites, the amino sequences of core and NS5B were uploaded on the online available tool SignlN-PP. The predicted sites crossing the threshold were counted and compared among the two study groups.

## Abbreviations

ALT: Alanine amino transferases
AST: Aspartate amino transferases
ELISA: Enzyme linked immunosorbant assay
HCV: Hepatitis C virus
IFN: Interferon
PegIFN: Pegylated Interferon
LDL: Low density lipoprotein
RNA: Ribonucleic acid
PCR: Polymerase chain reaction.
